# Quantitative and Qualitative Evaluation of Enamel Erosion Following Air Abrasion with Bioactive Glass 45S5

**DOI:** 10.3290/j.ohpd.a44689

**Published:** 2020-07-04

**Authors:** Dimitrios Dionysopoulos, Kosmas Tolidis, Effrosyni Tsitrou, Pantelis Kouros, Olga Naka

**Affiliations:** a Assistant Professor, Department of Operative Dentistry, School of Dentistry, Aristotle University of Thessaloniki, Thessaloniki, Greece. Idea, hypothesis, experimental design, performed the experiment, wrote the manuscript.; b Professor, Department of Operative Dentistry, School of Dentistry, Aristotle University of Thessaloniki, Thessaloniki, Greece. Idea, experimental design, proofread the manuscript.; c Associate Professor, Department of Operative Dentistry, School of Dentistry, Aristotle University of Thessaloniki, Thessaloniki, Greece. Idea, experimental design, proofread the manuscript.; d Assistant Professor, Department of Operative Dentistry, School of Dentistry, Aristotle University of Thessaloniki, Thessaloniki, Greece. Performed the experiment, proofread the manuscript.; e Assistant Professor, Department of Prosthodontics, School of Dentistry, Aristotle University of Thessaloniki, Thessaloniki, Greece. Performed the experiment, wrote the manuscript.

**Keywords:** air abrasion, bioglass, enamel erosion, scanning electron microscopy, surface loss

## Abstract

**Purpose:**

To evaluate the effect of pre-treatment air abrasion of surfaces using bioactive glass 45S5 on the progression of erosion in bovine enamel induced by a common soft drink.

**Materials and Methods:**

Twelve intact bovine incisors were selected and 24 enamel samples were prepared and randomly assigned to two groups (n = 12): 1. control group, no anti-erosive treatment; 2. experimental group: samples were air abraded with bioglass 45S5 before the erosive challenge. The enamel samples were submitted to erosive cycling using a common soft drink. Enamel surface loss was evaluated using optical profilometry; surface microhardness and roughness changes were determined using Vickers method and Vertical Scanning Interferometry, respectively. In addition, SEM observations and EDS analysis were performed to detect any alterations in surface morphology and mineral content. The data were statistically analysed using one-way ANOVA and Tukey’s post-hoc test at a significance level of α = 0.05.

**Results:**

The experimental group exhibited less (18.7%) surface loss than did the control group (p < 0.05), while also presenting a statistically significantly smaller decrease in surface microhardness compared to the control group after erosive cycling (p < 0.05). However, neither group showed a statistically significant change in surface roughness (p > 0.05). After the treatments, changes in surface morphology and mineral content of enamel were observed.

**Conclusions:**

Surface pre-treatment using air abrasion bioglass 45S5 may help prevent enamel erosion induced by excessive consumption of soft drinks. Further clinical trials are needed to confirm the effectiveness of this method and its clinical significance.

Dental erosion is a pathological, long-term process which leads to loss of dental hard tissues. It is attributed to acidic and chelating agents of non-microbiological origin which repeatedly interact with tooth surfaces. Various chemical (e.g. pH and buffering capacity of the diet), biological (e.g. saliva, pellicle, composition and structure of the teeth) and behavioral (e.g. number of meals, dental hygiene, sports, alcohol, soft drinks, toothbrushing) factors play a role in the pathogenesis of dental erosion.^20^

Today, the daily consumption of carbonated soft drinks (which often have a very low pH) has become widespread, especially in children, and may lead to dental erosion.^35^ When dental tissues undergo erosive attack, the consequences may include reduction of surface microhardness due to dissolution of mineral compounds,^33^ alterations in surface roughness,^16^ changes in surface morphology,^5^ and eventually, surface loss of tooth tissues.^25^

The structural changes of tooth tissues induced by erosive agents can be evaluated by various methods. Quantitatively, dental erosion is estimated by measuring surface loss of enamel or dentin. The most common methods of measuring tooth surface loss are profilometry^15^ and confocal laser scanning microscopy (CLSM).^21,23^ Changes in surface hardness, roughness, morphology, and mineral composition of tooth tissues after an acid attack are usually recorded to qualitatively evaluate dental erosion. In particular, tooth surface microhardness changes can be evaluated using Vickers^2^ or Knoop^25^ methods with a microhardness tester. Surface roughness alterations can be measured using profilometry^30^ or atomic force microscopy (AFM),^35^ surface morphology alterations can be observed using SEM,^28^ AFM,^27^ or CLSM.^21^ Changes in mineral composition can be detected by energy dispersive X-ray spectroscopy (EDS),^8^ Fourier transform infrared spectroscopy (FTIR),^28^ or FT Raman spectroscopy.^5^

The literature mentions multiple clinical techniques which have been suggested for preventing the destructive effect of erosive agents on dentition. These techniques are effective via two mechanisms: by modifying the surface of hard tissues so that it becomes more insoluble in acid, or by creating a protective layer on the surface of the hard dental tissues, which limits the destructive effect. Methods acting via the first mechanism mainly include laser irradiation using different laser wavelengths, such as carbon dioxide (10.6 μm),^13^ Er,Cr:YSGG (2.780 nm),^11^ Er:YAG (2.940 nm),^29^ and Nd:YAG (1.064 nm).^26^ The most common methods acting through the second mechanism are the use of stannous fluoride (SnF_2_) products, which deposit a barrier layer onto the pellicle-coated surface and strongly binds onto tooth surfaces,^14^ and bioactive glass treatment which, after interaction with saliva in acidic conditions, forms a protective layer rich in Ca, P, and Si ready to form hydroxyapatite.^2^

Generally, bioactive glasses are surface-reactive glass-ceramic biomaterials and include bioactive glass 45S5. This is an inorganic, amorphous calcium-sodium-phosphosilicate compound which has been broadly used in dental applications due to its bioactive properties. It can interact with saliva to form a hydroxycarbonate apatite layer, which is chemically bonded to the dental tissues.^7^

The aim of this in vitro investigation was to evaluate the influence of air-abrasion surface pre-treatment with bioactive glass 45S5 on bovine enamel erosion induced by a common soft drink. Dental erosion was quantitatively estimated by measuring the depth of the erosive lesions using optical profilometry. Erosion was qualitatively assessed by measuring Vickers surface microhardness and surface roughness using vertical scanning interferometry (VSI). Additionally, SEM and EDS were also used to examine the surface morphology and mineral content alterations, respectively, after treatment.

The null hypotheses were: 1. there would be no statistically significant differences in surface loss between the two experimental groups after erosive cycling; 2. no statistically significant differences would exist in surface microhardness decrease between the two experimental groups after erosive cycling; 3. there would be no statistically significant differences in surface roughness change between the two experimental groups after erosive cycling.

## Materials and Methods

### Enamel Samples

Twelve intact bovine incisors were selected in the current investigation. The teeth were stored in a 0.5% chloramine-T solution at 6°C for up to 1 month prior to the experiment. The crowns of the teeth were separated from the roots, and each crown was cut in the middle using a water-cooled diamond disk (Isomet, Buehler; Lake Bluff, IL, USA). Each specimen was approximately 4 mm long and 4 mm wide. The 24 prepared specimens were observed using an optical microscope to confirm their structural integrity, then randomly divided into two groups of 12 specimens each (n = 12). Subsequently, the specimens were embedded in epoxy resin (Epofix resin, Struers; Denmark, Copenhagen) and the enamel surfaces were ground and sequentially polished on a polishing machine (Jean Wirtz TG 250; Dusseldorf, Germany) using 600-, 800-, 1000-, and 1200-grit silicon carbide abrasive papers (Struers). Immediately after polishing, the specimens were immersed in an ultrasonic bath (Euronda Spa; Vicenza, Italy) for 5 min and stored in a remineralising solution for 24 h at 37°C before the experiment. The composition of the remineralising solution is described in Dionysopoulos et al^9^ and Table 1.

**Table 1:** Technical characteristics of the materials used

Product	Manufacturer	Composition	Lot number
ProSylc	Velopex; Harlesden, UK	100% NovaMin Particle size: 30-60-90 μm SiO_2_: 45%, CaO: 24.4%, Na_2_O: 24.6%, P_2_O_5_: 6%	160316
Coca Cola	Coca Cola, 3E Company; Thessaloniki, Greece	Water, sugar, carbon dioxide, caramel color E 150d, phosphoric acid, natural flavors, caffeine. pH = 2.47	–
Remineralisation solution	–	0.103 g/l of CaCl_2_, 0.019 g/l MgCl_2_•6H_2_O, 0.544 g/l KH_2_PO_4_, 2.24 g/l KCl and buffer (TCP-KOH). pH=7	–

### Experimental Groups

The enamel samples of group 1 (control) did not receive any anti-erosive treatment during the experiment. The surface of group-2 specimens was air abraded with bioactive glass particles before the erosive challenge. Specifically, an Aquacare clinical air-abrasion unit (Velopex; Harlesden, UK) was used to treat the enamel surface of the specimens with ProSylc (Velopex), which contains bioactive glass 45S5 powder (NovaMin), employing the following operating parameters (Milly et al^22^): air pressure: 20 psi (ca 1.38 bar); powder flow rate dial: 1 g/min; nozzle angle: 90 degrees; nozzle-surface distance: 5 mm; internal nozzle diameter: 900 μm: duration of air abrasion: 10 s. The composition of ProSylc is presented in Table 1.

### Erosive Challenge

A common soft drink (Coca Cola, 3E Company; Thessaloniki, Greece) was used as the erosive agent (Table 1). The pH of the soft drink was evaluated using a digital pH meter (Orion StarSeries ISE Meter, Thermo Scientific; Beverly, MA, USA) and was found to be stable (pH = 2.47 ± 0.09) for the duration of the experiment at room temperature (23 ± 1°C). Each sample was rinsed with distilled water for 10 s, then immersed in 6 ml of fresh soft drink in a plastic container 4 times of 2 min. The specimens were then rinsed again with distilled water and stored in fresh remineralising solution. This cycling procedure was carried out at 0, 12, 24, 36, 48, and 60 h. The protocol of the erosive challenge followed Wang et al.^35^

### Surface Loss Evaluation

Enamel loss was evaluated after the erosive challenge by white-light optical profilometry (Bruker, ContourGT; Berlin, Germany). Before erosive cycling, half of each sample’s surface was covered with one-sided silver adhesive tape (Wonder Tape, Achem Technologies; Taipei City, Taiwan). After erosive cycling, the tape was removed, and four images (20X magnification) of the center of the enamel surface were taken. Each image corresponded to a surface of 0.317 x 0.238 mm.^2^ Enamel surface loss was calculated after superimposing the baseline and post-erosion profiles.

### Surface Microhardness Evaluation

Enamel surface microhardness was evaluated before and after erosive cycling using a Vickers hardness tester (HMV-2000, Shimadzu; Tokyo, Japan) with a load of 200 g and indentation time at 10 s. Five indentations were performed on the top surface of each specimen: one in the centre, and one in every quadrant (500 μm apart). The indentation dimensions were measured using the hardness tester’s optical microscope, and data were independently averaged and reported as Vickers Hardness Numbers (VHN).

### Evaluation of Surface Roughness

The measurements were performed before and after erosive cycling using a vertical scanning interferometer (ContourGT, Bruker). Surface roughness analysis of the enamel specimens was performed according to ISO 25178 (non-contact type). Three images were obtained (magnification 20X) from each specimen in each quadrant of the surface, which corresponds to a surface area of 0.317 x 0.238 mm^2^. Vision64 software (ContourGT, Bruker) was used to acquire the data and compute the mean surface roughness in Sq units of each image. The mean was calculated of the values of the twelve images of each specimen.

### SEM and EDS Analysis

Changes of surface morphology after air abrasion and erosive challenge were observed using SEM. Three samples of each experimental group before and after erosive cycling were prepared and mounted on aluminum stubs, sputter coated with carbon to a thickness of approximately 200 Å in a vacuum evaporator (at low vacuum), and observed using SEM (JSM-840, JEOL; Tokyo, Japan) at an accelerating voltage of 20 KV and working distance of 10 mm. Five SEM images were obtained from the surface of the samples (500X magnification) for investigating surface morphology. EDS was applied to the same images in order to evaluate changes in mineral composition of the enamel surfaces after the respective treatments.

### Statistical Analysis

The outcomes of the study were statistically analysed using SPSS Statistics 20.0 software (IBM; Chicago, IL, USA). Data were preliminarily tested for normality and homogeneity using the Shapiro-Wilk test and Levene’s test, respectively. Surface loss, surface microhardness, and surface roughness data of the enamel specimens were statistically analysed using one-way ANOVA; Tukey’s post-hoc test was used to detect statistical differences at α = 0.05. Mineral composition of the enamel surface was analysed using Wilcoxon signed rank and Kruskal-Wallis tests, with significance preset at α = 0.05.

## Results

### Surface Loss

**Fig 1:**
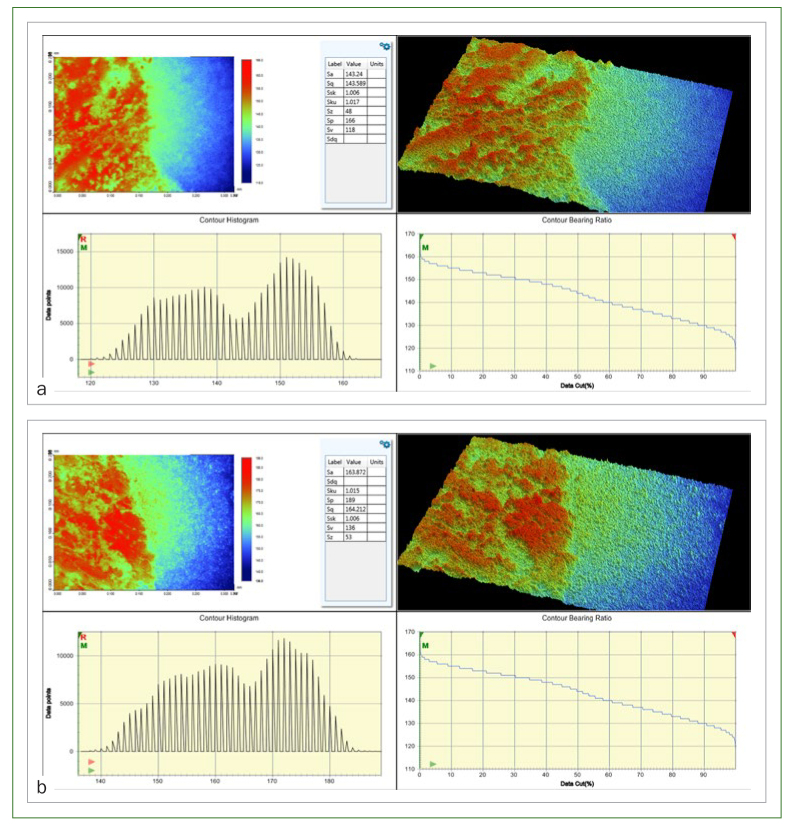
Representative topographic maps and surface analysis of enamel specimens of the experimental groups (20X magnification) at the center of the specimens, showing the depth of the erosive lesions. The contour histogram and the contour bearing ratio are also shown. a: group 1 (control); b: group 2 (surface air abraded with bioglass).

Means (± SD) of surface loss (μm) of the experimental groups after erosive challenge are presented in Table 2. Representative topographic surface maps (magnification 20X) and surface analysis of the experimental groups are illustrated in Fig 1. Surface loss was detected after erosive challenge. Air-abrasion pre-treatment with bioglass particles significantly reduced the surface loss in comparison with the control group (p < 0.01).

**Table 2:** Means and standard deviations of surface loss (μm) of the experimental groups after erosive challenge, anddecrease in surface loss of the tested treatment vs the control group

Groups	Treatments	Mean surface loss	% decrease loss compared to control
1	Control	65.8 ± 9.0^A^	–
2	Air abrasion with bioglass	53.5 ± 8.5^B^	18.7%

Different superscript uppercase superscripts in columns indicate statistically significant differences between the two groups (p < 0.05).

### Surface Microhardness

Means (± SD) of surface microhardness in VHN of the experimental groups before and after erosive challenge are presented in Table 3. Surface microhardness was significantly reduced after erosive challenge (p < 0.05). Air-abrasion pre-treatment with bioglass exhibited significantly less decrease in surface microhardness compared to the control group (p < 0.001).

**Table 3:** Means and standard deviations of surface microhardness (VHN) of the experimental groups before and after the erosive challenge

Groups	Treatments	Before erosive challenge	After erosive challenge	ΔVHN	%VHN decrease
1	Control	277.2 ± 19.5^Aa^	157.4 ± 21.7^Ab^	119.8 ± 24.4^A^	43.1%
2	Air abrasion with bioglass	269.8 ± 23.0^Aa^	208.5 ± 23.6^Bb^	61.3 ± 19.2^B^	21.9%

ΔVHN: reduction of surface mirohardness in VHN after the erosive challenge. %VHN decrease: % decrease of surface microhardness after erosive challenge. Same superscript uppercase letters in columns indicate no statistically significant differences between groups (p > 0.05). Same superscript lowercase letters in rows indicate no statistically significant differences between values before and after erosive challenge (p > 0.05).

### Surface Roughness

Means (± SD) of surface roughness (Sq, μm) of the experimental groups before and after erosive challenge are shown in Table 4. Surface roughness did not change after erosive challenge (p > 0.05). Air-abrasion pre-treatment with bioglass did not induce significantly different surface roughness compared to the control group (p = 0.65).

**Table 4:** Means and standard deviations of surface roughness (Sq, μm) of the experimental groups before and after the erosive challenge

Groups	Treatments	Before erosive challenge	After erosive challenge	ΔSq	%Sq increase
1	Control	0.215 ± 0.010^Aa^	0.221 ± 0.006^Aa^	0.006 ± 0.010^A^	2.7%
2	Air abrasion with bioglass	0.216 ± 0.007^Aa^	0.218 ± 0.006^Aa^	0.002 ± 0.001^A^	0.9%

ΔSq: increase of surface roughness in Sq after the erosive challenge, %Sq increase: % increase of surface roughness after erosive challenge. Same uppercase superscript letters in columns indicate no statistically significant differences between the groups (p > 0.05). Same lowercase superscript letters in rows indicate no statistically significant differences between values before and after erosive challenge (p > 0.05).

### SEM Observations

**Fig 2:**
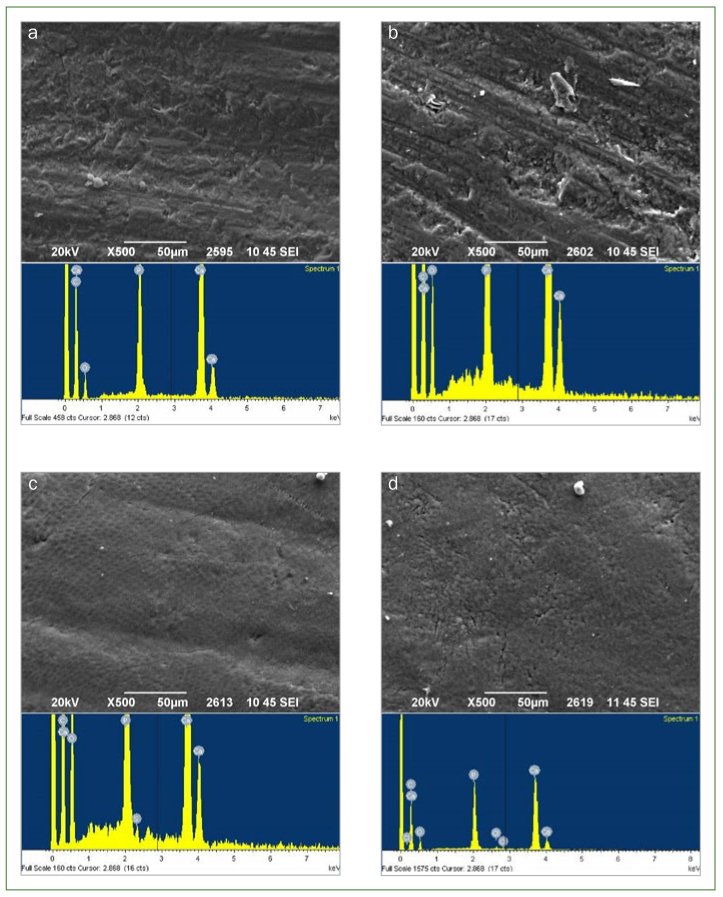
Representative SEM images of the enamel surface of the experimental groups (magnification 500X) showing morphological changes before and after the erosive challenge. The EDS spectrum of each element of the enamel surface appears below the SEM images. a: group 1 before erosive challenge; b: group 2 before erosive challenge; c: group 1 after erosive challenge; d: group 2 after erosive challenge.

Representative SEM photomicrographs of the enamel surface of the experimental groups before and after erosive challenge are shown in Fig 2. SEM images revealed alterations in enamel surface morphology in group 2 after air-abrasion surface pre-treatment with bioglass particles. More specifically, deposits of spherical inorganic particles were detected on the surface, as shown in Fig 2b. Furthermore, after the erosive challenge, more clear enamel surfaces were observed, in which enamel prisms were visible (Figs 2c and 2d).

### EDS Analysis of Mineral Content

The EDS spectrum of each representative photomicrograph of enamel surfaces is presented below the SEM images (Fig 2). For each experimental group, the contents (wt%) of each element of the enamel surface before and after erosive challenge are presented in Table 5. EDS analysis revealed an increase in silica (Si) after air-abrasion pre-treatment, indicating the existence of bioglass particles on the enamel surface. In addition, following the erosive challenge, the control group exhibited a decrease in Ca and P on the enamel surface (p < 0.05). In contrast, the specimens that were air abraded with bioglass did not present changes in calcium and phosphorus content after erosive challenge.

**Table 5:** Means and standard deviations of elemental content (%wt) of enamel surface before and after the erosive challenge of the experimental groups

Elements	Before erosive challenge		After erosive challenge	
	Group 1	Group 2	Group 1	Group 2
Ca	34.66 ± 3.57^a^	36.84 ± 4.11^a^	29.05 ± 3.17^b^	41.99 ± 4.22c
P	22.88 ± 2.68^a^	21.81 ± 2.13^a^	17.88 ± 2.80^b^	24.74 ± 2.52^a^
Si	0.00 ± 0.00^a^	0.35 ± 0.08^b^	0.00 ± 0.00^a^	0.21 ± 0.05^c^
Na	0.46 ± 0.10^a^	0.43 ± 0.12^a^	0.48 ± 0.10^a^	0.20 ± 0.06^b^
Cl	0.17 ± 0.04^a^	0.38 ± 0.08^bc^	0.34 ± 0.07^b^	0.42 ± 0.12^c^
F	0.18 ± 0.05^a^	0.34 ± 0.09^b^	0.20 ± 0.05^a^	0.19 ± 0.07^a^
O	43.66 ± 4.16^a^	40.33 ± 4.28^a^	52.96 ± 5.82^b^	34.21 ± 3.76^c^

Different superscript lowercase letters in rows indicate statistically significant differences (p < 0.05).

## Discussion

According to the results of the present study, the first null hypothesis – that there would be no statistically significant differences in surface loss between the two experimental groups after the erosive challenge – was rejected. Although enamel surface loss was observed in both experimental groups after the erosive challenge, the specimens air abraded with bioactive glass presented less surface loss (18.7%) than did the control group specimens. This means that the tested preventive treatment protected against dental erosion. This finding is in agreement with previous investigations concerning anti-erosive activities of bioactive glass treatments.^2,18^

The protective action of bioactive glass involves the initiation of a series of chemical reactions with the tooth surface and saliva under acidic conditions, leading to the formation of a hydroxycarbonate apatite layer which is chemically bonded to the tooth surface.^17^ This layer acts as a protective barrier to erosive attacks, and may reduce the dissolution of tooth tissues. Moreover, this layer is rich in calcium and phosphate ions ready to form hydroxyapatite under acidic conditions, and provides buffering and remineralisation.^2^ Additionally, Bakry et al^3^ reported that enamel specimens treated with bioglass 45S5 paste were completely covered with a layer of brushite crystals, which was resistant to brushing-abrasion challenge, and that these crystals converted to hydroxyapatite crystals when stored in artificial saliva for 14 days. This bioactive behaviour may facilitate restoration of incipient enamel erosive or caries lesions.

Due to its simplicity and accuracy, optical profilometry is a very common method to evaluate enamel surface loss after erosive challenge. It has been used in previous studies to evaluate tooth surface loss following various erosive protocols.^15,16,18,22^ A profilometer is an instrument used to measure the profile of a surface, in order to quantify its surface roughness. Additionally, critical dimensions such as step, curvature, and flatness are computed from the surface topography.^31^

Based on the results of the current investigation, the second null hypothesis – that there were no statistically significant differences in surface microhardness decrease between the two experimental groups after the erosive challenge – was rejected. This coincides with the results of previous studies which revealed less surface microhardness reduction of the enamel after application of bioglass 45S5.^12,32^ Reduction in surface hardness could be explained by mineral loss of the enamel surface due to the acidic and chelating activities of soft drink components.^35^ The protective action of bioglass 45S5 treatment may be explained by the remineralising effects that take place and the protective layer formed, as mentioned above.

Tooth surface hardness reflects the structure and composition of the tissue. Because the tooth surface contains high amounts of inorganic compounds and is rigid when intact, surface hardness is also high. Consequently, measuring changes in tooth surface hardness after application of an erosive agent demonstrates the influence of acid on this surface. In the present study, we used the Vickers method to evaluate the changes of enamel microhardness after the erosive challenge. This method has been used in many previous investigations for the same purpose.^2,11,35^

The third null hypothesis – that there would be no statistically significant differences in surface roughness change between the two experimental groups after the erosive challenge – was accepted. The two experimental groups presented a slight increase in enamel surface roughness after erosive challenge, but this increase was not statistically significant. It has been found that changes in surface roughness, which take place during early erosive attack due to removal of calcium and phosphate ions from the enamel surface, occur relatively quickly.^1^ Mylonas et al^24^ demonstrated that surface roughness of natural enamel surfaces decreased as acid immersion time increased, indicating smoothening of the aprismatic enamel surface. On the other hand, polished enamel specimens presented rougher surfaces with increasing erosion. This corroborates with clinical observations that patients who present erosive tooth wear exhibit natural enamel surfaces that are smoother and shinier than healthy surfaces due to loss of surface structure and texture.^4^

Concerning enamel surface roughness change after air-abrasion pre-treatment, a previous study^22^ found that the same pre-conditioning used here increased the average surface roughness of enamel. However, in the present investigation, this preventive technique did not significantly increase the enamel surface roughness. This difference may be explained by the different composition of the bioglass powder used in air abrasion.

Surface roughness of a material is quantified by the deviations of the direction of a normal vector of a real surface from its ideal form. Generally, a rough surface has large deviations, while a smooth surface has small deviations. Changes in surface roughness of tooth tissues after an acidic attack reflect the interaction of the erosive agents with tooth structures. This interaction may lead to removal of inorganic compounds or modification of hydroxyapatite crystals of the tooth surface, and depends mainly on the duration of the interaction, the acidity of the erosive agent, the oral environmental conditions, and the tooth substrate. The areal roughness parameters are defined in the ISO 25178 series, and the most common for evaluation of tooth surface roughness are the arithmetical mean height of the peaks of the surface area (Sa) and the root mean square height of the peaks of the surface area (Sq).^10,19^

In the present study, the interaction between tooth tissues and erosive solution did not influence enamel surface roughness in either experimental group. Previous studies have obtained contradictory results regarding changes in surface roughness after application of different erosive challenges, due to the various aforementioned factors that affect surface texture. One study reported an increase in surface roughness after erosive challenge;^16^ another reported a decrease in surface roughness,^24^ whereas a third reported no change.^11^ This is the reason that surface roughness changes are recorded only for evaluating qualitative characteristics of dental erosion and because they do not provide information about the progress of erosive tooth wear.

Early changes of dental erosion usually include initial breakdown of prism-interprism interfaces, further increasing size of the prisms relative to their initial size, loss of superficial and deeper topographical features, which are attributed to short-term activity of an erosive agent.^24^ In the present study, SEM images obtained after the aggressive erosive challenge showed smoother enamel surfaces in which the enamel prisms were visible. These surfaces are more susceptible to further progression of erosive wear than the intact enamel surfaces which contain a hypermineralised superficial layer that retards the progression of the erosion.^6^

After air-abrasion pre-treatment with bioglass, SEM observations revealed spherical compounds covering the entire enamel surface. These compounds were identified by EDS as bioglass particles containg silica. Previous studies reported that bioglass application on eroded enamel surfaces could, under acidic conditions, form a crystalline layer rich in calcium and phosphate, which might protect against enamel erosion.^2^ In the present study, the specimens which received bioglass pre-treatment showed reduced erosive activity of the soft drink, which may be due to the existence of this protective layer.

Generally, human teeth are regarded as the most appropriate source in terms of clinical relevance. However, their composition is not homogeneous, due to variations in genetic factors, environmental conditions, diet and age, which may lead to differences in their response under erosive challenges. In contrast, bovine teeth have a more uniform composition when compared to human teeth, thus providing a less variable response to erosive treatments. Although bovine enamel is more porous than human enamel, resulting in faster demineralisation and remineralisation, these discrepancies are quantitative, not qualitative. Additionally, the erosive lesions produced in bovine teeth have a mineral distribution and structure that resembles lesions produced in human teeth, both in enamel and dentin. As a result, bovine teeth can be considered an acceptable alternative to human teeth and are broadly used in dental erosion studies.^6,11,23^

## Conclusions

Considering the limitations of the study, air-abrasion pre-treatment with bioglass 45S5 may prevent enamel erosion induced by excessive consumption of soft drinks. In the present investigation, the tested treatment quantitatively and qualitatively influenced enamel erosion after erosive challenge. Thus, this treatment should be added to an individually tailored preventive programme, together with measures such as diet modification, oral hygiene education, use of anti-erosive agents, and regular surveillance. Clinical trials are necessary to confirm the protective action of this anti-erosive treatment and to evaluate its clinical significance.
